# Noninvasive Assessment of Fibrosis in Patients with Nonalcoholic Fatty Liver Disease

**DOI:** 10.1155/2015/343828

**Published:** 2015-04-30

**Authors:** Elena Buzzetti, Rosa Lombardi, Laura De Luca, Emmanuel A. Tsochatzis

**Affiliations:** UCL Institute for Liver and Digestive Health, Royal Free Hospital and UCL, London NW3 2QG, UK

## Abstract

Nonalcoholic fatty liver disease (NAFLD) is prevalent in 20–25% of the general population and is associated with metabolic risk factors such as obesity, diabetes mellitus, and dyslipidemia. Histologically, NAFLD ranges from simple steatosis to nonalcoholic steatohepatitis (NASH), fibrosis, and cirrhosis. As NASH develops in only 10–15% of patients with NAFLD, it is not practical to biopsy all patients who present with NAFLD. Noninvasive fibrosis tests have been extensively developed recently and offer alternatives for staging fibrosis. Despite their increasing use, such tests cannot adequately differentiate simple steatosis from NASH. At present, such tests can be used as first line tests to rule out patients without advanced fibrosis and thus prevent unnecessary secondary care referrals in a significant number of patients. In this review we present the evidence for the use of noninvasive fibrosis tests in patients with NAFLD.

## 1. Introduction

Nonalcoholic fatty liver disease (NAFLD) is the most common cause of abnormal liver function tests in primary care in industrialized countries. It is associated with insulin resistance (IR) and is considered as the hepatic manifestation of the metabolic syndrome (MeS) [1].

The reported prevalence of NAFLD varies widely depending on the studied population, the diagnostic modality, and the definition [2]. According to the latest data, 20–30% of European and Middle East population have NAFLD, with an incidence of 39/100.000 inhabitants in the UK [3]. In USA the prevalence of NAFLD as assessed by MR spectroscopy is 34%, while it is lower in the Far East (15%) [4].

Ultrasonography, in association with routine laboratory investigations, is an acceptable first line test to identify patients with NAFLD, even if it has suboptimal sensitivity and reproducibility, being an operator-dependent technique [4].

From a histological point of view, NAFLD encompasses a wide spectrum of liver pathology, from simple steatosis to steatohepatitis, fibrosis, and cirrhosis [5]. While simple steatosis is associated with a low risk of progression, patients with NASH, estimated to be 15–20% of the NAFLD population, have increased risk of liver-related morbidity and mortality from cirrhosis and development of HCC [6, 7]. Other metabolic risk factors, such as the presence of metabolic syndrome, are associated with more severe liver disease [8].

The pivotal issue in the management of patients with NAFLD is the diagnosis of steatohepatitis and fibrosis at an early stage. This would allow the identification of those patients who require stricter follow-up, targeted lifestyle interventions for weight reduction, management of the components of the metabolic syndrome, and eventual inclusion in trials of new treatment strategies [9].

Liver biopsy is the gold standard for diagnosing NASH and assessing fibrosis, though its limitations are well known: potential risk of sampling errors, intra- and interobserver variability, invasiveness, and scarce tolerability by the patient [10, 11]. These characteristics, together with the high prevalence of hepatic steatosis and its low risk of progression in the majority of affected people, make liver biopsy an inappropriate first line tool for diagnosis in unselected patients.

Noninvasive liver fibrosis tests have been extensively developed recently and offer alternatives for staging fibrosis. In this review, we discuss the evidence for their use in patients with NAFLD.

## 2. Noninvasive Fibrosis Tests

In the last years, we have witnessed explosive development and use of noninvasive fibrosis tests (Table 1). These can be divided into three categories, namely, simple or indirect serum markers, direct serum markers, and imaging modalities [13].

(i) Indirect or class II serum markers consist of routine biochemical tests such as transaminases, platelet count, and albumin that are usually combined with demographic characteristics potentially linked to liver fibrosis such as age or diabetes [14].

Indirect serum markers and panels usually have dual cut-offs: a high cut-off with high specificity and a low cut-off with high sensitivity. If these cut-offs are combined, depending on the clinical scenario and prevalence of the examined disease, the numbers of false positive and false negatives are minimized. However, a proportion of patients will fall in the indeterminate range of values and will therefore need further testing.

(ii) Direct or class I serum markers detect the fibrogenic process and the extracellular matrix turnover. Such markers include hyaluronic acid, collagenases and their inhibitors (TIMP), and profibrotic cytokines (TGF beta) [15]. Although they perform better than indirect serum markers, their sensitivity is low in the initial stages of fibrosis and they are not routinely available in most clinical laboratories [14].

(iii) Imaging modalities measure the elasticity and/or stiffness of the liver tissue with MR and US techniques.

Transient elastography (Fibroscan, Echosens, Paris, France) is a noninvasive US-based technique in which a low-frequency (50 Hz) elastic shear wave is generated by a transducer and then propagates through the tissues. The speed of propagation is proportional to the stiffness of the crossed tissue and its measurement can, through specific software, provide values of liver stiffness expressed in kilopascal (kPa) [16]. The final value is the median of 10 valid measurements.

TE is painless and rapid and thus highly acceptable to patients. The volume of liver studied with this method is a cylinder of 1 × 4 cm length, therefore 100 times bigger than a sample taken by liver biopsy.

TE has already been incorporated in the international guidelines for CHB and CHC for pretreatment fibrosis assessment [17, 18] and it has also been evaluated as a potential technique to predict the presence and degree of portal hypertension [19, 20] and HCC [21].

In ARFI (acoustic radiation force impulse), the elastography system is integrated on an ultrasound machine, allowing the operator to select the site of liver stiffness measurement (LSM) through a real-time B-mode ultrasonography and localize the shear wave avoiding vessels and ribs.

This technique showed a similar accuracy when compared to Fibroscan [22–24] in detecting fibrosis and/or cirrhosis. The range of measured values is narrower than Fibroscan, while quality criteria for ARFI have not yet been established.

The supersonic shear imaging (SSI) or Shear Wave Elastography is also built on a US device but, while ARFI is based on the emission of a single pulse wave, in SSI the transducer emits several wave fronts at increasing depth, with a frequency band ranging from 60 to 6000 Hz.

It generates real-time color mapping of the elasticity, which is integrated in a standard B-mode image, allowing a quantitative representation of the tissue elasticity. The final value will be the average of many obtained by selecting the region of measurement with guidance from both the B-mode and SWE images [25].

Magnetic resonance elastography (MRE) combines the ability of elastography to provide information about liver tissue texture to the advantages of MR, which allows the assessment of the entire liver rather than a small part [26].

The main limitation of all the above noninvasive liver fibrosis tests is the absence of uniformly established and validated cut-offs for different disease etiologies and fibrosis stages.

## 3. Noninvasive Diagnosis of NASH

### 3.1. Liver Enzymes

Both serum levels of aspartate aminotransferase (AST) and alanine aminotransferase (ALT) may increase in patients with NAFLD and this is more prominent for ALT rather than AST [27]. However, the increase is not proportionate to the liver inflammation or fibrosis and it is well documented that patients with significant fibrosis may present with normal transaminases [28].

Data from different studies report a low accuracy for ALT levels in predicting NASH, with an area under the curve (AUC) of 0.6 [29, 30]. Reducing the upper limit of normal cut-off value for ALT from 30 to 19 U/L showed an improvement of sensitivity in a cohort of 233 patients undergoing bariatric surgery, but at the expense of specificity [31].

### 3.2. Cytokeratin 18

In the last ten years, emerging data suggested that hepatocellular apoptosis, a genetically determined form of cell death, may have a significant role in the transition from simple steatosis to NASH [32]. Within the apoptotic process, various caspases are activated with the subsequent cleavage of different substrates. The main substrate in the liver is cytokeratin 18 (CK-18), which is an intermediate filament protein.

In total there have been more than eight studies exploring the diagnostic accuracy of CK-18 fragments levels in NASH. The main issue is that the cut-offs for diagnosis of NASH varied among studies; therefore this remains an unvalidated test [6]. In a cohort of 139 patients with biopsy-proved NAFLD compared to 155 healthy controls, CK-18 fragments levels independently predicted the presence of NASH (AUC 0.83) with a sensitivity of 0.75 and specificity of 0.9 [33]. Similar results were obtained by the same group in a cohort of 201 pediatric patients [34].

In a recent study, involving a multiethnic population of 424 patients, CK-18 fragments levels showed a high specificity for NAFLD and fibrosis but a limited sensitivity/specificity for NASH (58%/68%) [35].

Therefore, CK-18 is not an adequate screening tool to discriminate NASH or early fibrosis in NAFLD patients.

### 3.3. PIIIPN

Terminal peptide of procollagen III (PIIIPN), involved in fibrogenic processes, was evaluated in a single validation study on 136 subjects with NAFLD (71 in validation cohort) as marker of NASH and developing fibrosis. Although its performance was promising, with an AUC of 0.85–0.87, further validation studies in larger cohorts are needed [36].

### 3.4. Panel Tests

NASH diagnostic is a proprietary test which encompasses serum levels of cleaved CK-18, adiponectin, and resistin. It was tested in a cohort of 101 patients that also included a validation group of 32 patients and showed a moderate reliability for discriminating NASH from simple steatosis (AUC of 0.85 and 0.73 in the derivation and validation cohort, resp.) [15].

The same panel was reevaluated in an independent sample of 79 patients and had a suboptimal AUC of 0.7. Thus, a new model was subsequently developed by the same group, namely, the NASH diagnostic panel that consists of presence of diabetes, gender, BMI, serum triglycerides, CK-18 fragments, and total CK-18 levels. Although the AUC was greater (0.8) than those for NASH diagnostics or CK-18 fragment levels alone (0.7 and 0.71, resp.) [37], this test needs further validation in independent cohorts.

The NashTest comprises 13 serum and clinical parameters, namely, total bilirubin, GGT, *α*2-macroglobulin, apolipoprotein A1, haptoglobin, age, gender, weight, height, AST, serum glucose, triglycerides, and cholesterol, and divides patients into three categories: not NASH, borderline NASH, and NASH [38]. NashTest showed a low sensitivity but high specificity in diagnosing NASH with an AUC ranging between 0.7 and 0.83 [38]. NashTest was also used in patients with hyperlipidaemia and morbid obesity and showed AUCs of 0.8 and 0.77, respectively, for the diagnosis of NASH [39, 40]. This test needs external validation in independent cohorts.

NAFIC score was derived and validated in a cohort of 117 and 442 Japanese biopsy-proven NAFLD patients [41]. The score, ranging from 0 to 4, was calculated considering cut-off values for serum ferritin, serum fasting insulin, and serum type collagen 7s. The AUC for predicting NASH was 0.85 and 0.78 in the estimation group and validation group, respectively [41].

In a backward validation study published in 2013, Nakamura et al. showed that the sensitivity of the NAFIC score for diagnosing NASH could further improve by adding different insulin levels (AUC 0.8, Se 72.0, Sp 62, PPV 62, and NPV 72%) [42]. Therefore, NAFIC score is promising but requires further validation.

### 3.5. Imaging Techniques

In a small study of 58 patients, magnetic resonance elastography (MRE) was highly accurate (AUC = 0.93) for discriminating patients with NASH from those with simple steatosis, with a sensitivity of 94% and a specificity of 73% [43]. Further validation is required, considering that MRE is costly and not widely available in routine clinical practice.

## 4. Noninvasive Staging of Liver Fibrosis

### 4.1. AST/ALT Ratio

The AST/ALT ratio is a simple noninvasive test that can detect cirrhosis with a reasonable accuracy as shown in patients with chronic hepatitis C [44]. Although data in patients with NAFLD are scarce, it is widely available and could be used in primary care. However, alcohol use should be excluded, as patients with alcoholic steatohepatitis would get a false positive result [45].

### 4.2. Ferritin

Ferritin is the primary tissue iron-storage in the liver but also an acute-phase protein; therefore it can be induced in both cases of iron overload and inflammatory systemic diseases [46].

Hyperferritinemia is prevalent in about 30% of subjects with NAFLD and can be the only altered laboratory parameter.

Whether high ferritin levels in patients with NAFLD are an expression of hepatic inflammation rather secondary to insulin resistance or represent true iron overload is still not clarified [47].

Indeed patients with NAFLD often have mild to moderate hepatic iron accumulation, and phlebotomies may improve insulin resistance [48–50].

Therefore, serum ferritin has also been proposed as a marker of both NASH and liver fibrosis.

In a study of 628 patients with NAFLD, serum ferritin levels greater than 1.5 times the upper value of normal were independent predictors of advanced fibrosis [51].

In another study of 482 patients with NAFLD, although hyperferritinemia was common, the extent of elevation did not correlate with the histological stage [52].

In a study of 1201 patients with biopsy-proven NAFLD, serum ferritin levels increased with increasing histological grade of steatosis, lobular inflammation, and ballooning; however its diagnostic performance for detecting presence of fibrosis was suboptimal (AUC of 0.6, 0.57, and 0.55 for detecting fibrosis, severe fibrosis, and advanced fibrosis, resp.) [53].

The above data suggest that serum ferritin cannot be used as a biomarker for NASH or fibrosis on its own; however it is incorporated in panels for liver fibrosis assessment as discussed later.

### 4.3. APRI

The aspartate aminotransferase to platelet ratio index (APRI) has been proposed as a simple test for staging fibrosis in several chronic liver diseases, firstly in CHC [54].

APRI has been designed to detect significant fibrosis (METAVIR ≥F2) and cirrhosis with dual cut-offs in both stages. Studies in patients with NAFLD are scarce compared to other diseases and have not always used the recommended cut-offs [55].

In a recent study involving 358 patients with biopsy-proven NAFLD, the sensitivity and specificity of APRI with a cut-off >1 for significant fibrosis were, respectively, 30 and 93% [56]. Therefore APRI, similar to other simple noninvasive panels, could be used to exclude significant fibrosis.

### 4.4. BARD

BARD score includes body mass index (BMI), AST/ALT ratio, and diabetes and has been designed to detect significant fibrosis (Brunt score ≥F3) [57].

Its main limitation is its high false positivity, based on the overestimation of BMI and presence of diabetes.

Using a cut-off point ≥2 the sensitivity for the detection of advanced fibrosis was between 86.8 and 100% while the specificity ranged from 32.5% to 34.7% in different studies [57–59].

Subsequently, an enhanced model of BARD was attempted by adding INR to the panel. This was tested in a small cohort of 107 patients and resulted in an improved composite score for sensitivity and specificity (AUC 0.88 versus 0.8) [59].

In a population of 242 NAFLD patients, the BARD score had the lowest specificity, sensitivity, Youden index, and predictive values for predicting both significant fibrosis and cirrhosis when compared with other (simple and complex) models for diagnosis of liver fibrosis [60].

### 4.5. NAFLD Fibrosis Score

NAFLD fibrosis score was constructed and validated in a population of 733 patients with NAFLD for detecting significant fibrosis (Brunt score ≥F3).

It consists of age, hyperglycemia, BMI, platelet count, albumin, and AST/ALT ratio and has dual cut-offs. The AUC for significant fibrosis is 0.88 and 0.82, with sensitivity of 81% and 77% and specificity of 77 and 71% applying the low cut-off score, and sensitivity of 51 and 43% and specificity of 98 and 96% applying the high cut-off score in the estimation and validation group, respectively [61]. Therefore, this score can be used to triage patients in primary care, as it accurately diagnoses patients without significant fibrosis.

### 4.6. FIB-4

The FIB-4 index was developed as a noninvasive panel to detect significant fibrosis in subjects with HIV-HCV coinfection and it has been independently validated in subjects with HCV infection [62, 63].

FIB-4 consists of age, AST, ALT, and platelet count. Similar to the NAFLD fibrosis score it has dual cut-offs, that is, a low cut-off of 1.45 with a high sensitivity and a high cut-off of 3.25 with a high specificity.

FIB-4 has showed a better accuracy in diagnosing advanced fibrosis in NAFLD patients when compared to other simple noninvasive tests in several studies (overall AUC 0.88) [64, 65].

Both the NAFLD fibrosis score and FIB-4 can be used in the primary care as triaging tests, in order to decide which patients do not have advanced fibrosis and therefore can be safely managed there.

### 4.7. Hepascore

Hepascore is a proprietary test that includes age, gender, bilirubin, gamma-glutamyl transferase (GGT), hyaluronic acid, and *α*2-macroglobulin [66].

This scoring system has been tested in NAFLD population in a study of 242 patients. In comparison with other serum biomarkers and complex scores [60] it apparently had a good diagnostic accuracy for staging fibrosis (AUC, sensitivity, and specificity of 0.73, 50.5%, and 88.3% for stage ≥F2, 0.81, 75.5%, and 84.1% for stage ≥F3, and 0.9, 87%, and 89% for cirrhosis, resp.). In another study it was showed that its reliability may vary depending on the influence of fasting on hyaluronic acid values [67].

### 4.8. ELF Test

The enhanced liver fibrosis (ELF) test is a simplified version of the original ELF (OELF) panel whose diagnostic performance has been validated for a variety of liver disorders [68].

ELF test is an algorithm that consists of hyaluronic acid (HA), amino-terminal propeptide of type III procollagen (PIIINP), and tissue inhibitor of metalloproteinase 1 (TIMP-1).

In the first validation study in a NAFLD/NASH population of 196 patients for the staging of liver fibrosis, the ELF panel had good performance in distinguishing severe fibrosis (stages 3-4) with an AUC of 0.9, but lower AUCs for moderate fibrosis and absence of fibrosis (0.82 and 0.76, resp.) [69]. Similar findings were confirmed in NAFLD pediatric populations [70, 71].

### 4.9. FibroTest and FibroMeter

FibroTest is a biomarker of liver fibrosis initially validated in patients with chronic hepatitis C [72]. It consists of bilirubin, haptoglobin, GGT, *α*2-macroglobulin, and apolipoprotein-A and classifies patients into 3 categories, namely, presence/absence of a certain stage of fibrosis or indeterminate values. Approximately a third of the patients have values in the indeterminate range [73].

The FibroMeter NAFLD score is a proprietary panel of serum markers that has shown a high diagnostic accuracy for the staging of fibrosis [74]. In a study of 235 NAFLD patients, the AUCs for the FibroMeter NAFLD score were 0.94 for significant fibrosis (≥F2), 0.93 for severe fibrosis (F3), and 0.9 for cirrhosis, with high sensitivity, specificity, PPV, and NPV. It is easily calculated from simple parameters and employing optimal cut-off values for the diagnosis of significant fibrosis, 97.4% of patients would be correctly classified [75].

All the above proprietary panels need independent validation by groups not involved in their creation and also in non-Caucasian populations.

### 4.10. Simple Scores versus Complex Scores

In a multicenter study of 242 patients, complex models (Hepascore, FibroTest, and FIB-4) were compared to simple models (APRI, BARD). For the prediction of significant fibrosis (≥F2) all these models had modest accuracy (AUC 0.71–0.74) with BARD being least accurate (AUC 0.61, *P* < 0.05 versus others), while Hepascore and FIB-4 had the highest specificity (88.3% and 87.5%, resp.). For the prediction of advanced fibrosis (≥F3), all complex models provided specificity values >80%, with Hepascore providing the highest sensitivity (75.5%), while all models (especially FIB-4 and Hepascore) had a good accuracy (AUC 0.8–0.86 versus 0.7, *P* < 0.05).

For the prediction of cirrhosis, Hepascore provided the highest sensitivity (87%). All models had reasonable specificity (>0.8) except for APRI. Complex scores had the best accuracy (AUC for Hepascore 0.94, FIB-4 and FibroTest 0.86) [60].

A recent meta-analysis has reported that FibroTest, ELF, and NAFLD fibrosis scores have significantly better diagnostic accuracy than BARD score and APRI, and their AUCs did not significantly differ from each other [6].

### 4.11. Imaging Techniques to Diagnose Fibrosis

Ultrasonography, CT scan, and MRI are commonly used in clinical practice for routine liver imaging. They can detect advanced cirrhosis with signs of portal hypertension (enlarged spleen and portal vein, and collateral venous circulation), but not fibrosis of lesser stages [76, 77].

Transient elastography (Fibroscan) is the most evaluated elastography-based technique for fibrosis assessment in NAFLD: it has been shown to be an accurate and reproducible methodology to discriminate patients without any degree of fibrosis from those with advanced fibrosis or cirrhosis both in adult and in pediatric NAFLD patients [78, 79].

A meta-analysis of 40 studies showed that elastography had good sensitivity and specificity for cirrhosis of different etiologies and less accuracy for lesser degrees of fibrosis but a precise validation of specific stiffness cut-off values for the various stages of fibrosis is still lacking [16].

Nevertheless this technique has some limitations. It can give falsely elevated values in certain conditions, such as acute hepatitis, extrahepatic cholestasis, hepatic congestion, congestive heart failure, hepatic amyloidosis, and recent food intake [80]. Moreover, using the conventional M probe, liver stiffness measurements are uninterpretable in 19% of the cases and this is mainly due to increased waist circumference and BMI, as it is frequently the case in patients with NAFLD [81].

A new dedicated XL probe for obese patients, which produces lower width shear waves, has been recently created. XL probe has similar overall diagnostic accuracy when compared to M probe with an AUC of 0.8–0.83 for fibrosis ≥F2, 0.85–0.87 for ≥F3, and 0.89–0.91 for cirrhosis [82, 83]. However, it generates lower stiffness values than the M probe; therefore different cut-offs should be used.

When using the M probe in all patients as first line and the XL probe in those failing the M probe measurements, 78–84% of patients will have reliable liver stiffness measurements [84].

In a recent study, liver stiffness was measured by Fibroscan, ARFI, and SSI and the results were compared with histological data. Pairwise comparison of AUCs values between SSI, Fibroscan, and ARFI showed no significant difference in diagnosing mild fibrosis (≥F1) or cirrhosis (F4). SSI showed higher accuracy in diagnosing significant fibrosis (≥F2) compared to ARFI and in diagnosing severe fibrosis (≥F3) compared to Fibroscan [23].

Real-time (RT) elastography is another technique of interest, as shown in a cohort of 181 patients, with a diagnostic accuracy of 0.83–0.96 depending on the assessed fibrosis stage [85].

In a recent retrospective analysis of 142 patients, MRE showed good accuracy for the diagnosis of advanced fibrosis (AUC 0.95) with high sensitivity and specificity (0.85 and 0.93, resp., at a cut-off of 4.15 kPa) [86]. These are probably the best results obtained by a noninvasive diagnostic technique for the present.

All elastography-based techniques need universally applicable validated cut-offs across different fibrosis stages. This would require adequately powered prospective studies.

## 5. Genetic Markers as Potential Future Noninvasive Prognostic Tests

It has also become increasingly apparent that genetic factors may influence the development and progression of NASH. These are polymorphisms of genes that regulate oxidative damage, the inflammatory cascade, and priming of fibrosis. The first such polymorphism described was a variant of patatin-like phospholipase-3 (PNPLA3) (also called adiponutrin), derived from an isoleucine-to-methionine substitution at residue 148 (single-nucleotide polymorphism or SNP). The GG genotype of PNPLA3 in patients with NAFLD has been associated with more severe steatosis, presence of NASH, and fibrosis [87, 88]. Furthermore, it seems to correlate with an increased risk of atherogenesis and subsequent cardiovascular events [89]. Carriers of the PNPLA3 rs738409 minor (G) allele have also a higher probability to develop NAFLD-correlated HCC, especially of the poorly differentiated type [90].

Another single nucleotide polymorphism which has been recently correlated with the development and prognosis of NAFLD is a glutamate to lysine amino acid substitution at residue 167 in transmembrane 6 superfamily member 2 (TM6SF2) sequence; the T allele of this gene is associated with the development of NAFLD, its progression to fibrosis and cirrhosis, and also altered cholesterol metabolism [91]; however there are some contradictions in literature and further studies are required to validate its role in larger cohorts of patients [92]. Further potential genetic determinants of NAFLD and progression of liver fibrosis are being evaluated in genome-wide association studies and may be used in the future as noninvasive prognostic indicators.

## 6. Conclusions

For the present several noninvasive diagnostic strategies have been proposed as alternatives to liver biopsy in patients with NAFLD, with various levels of diagnostic accuracy. Although the accurate diagnosis of NASH is still not possible with available noninvasive tools, there are several scores that can diagnose advanced fibrosis (≥F3). Simple panels, such as FIB-4 and NAFLD fibrosis score, are easily computable, have a high negative predictive value for advanced fibrosis, and have been validated against clinical outcomes. Therefore they could be used as first line test to “rule out” patients without advanced fibrosis and thus prevent unnecessary secondary care referrals in a significant number of patients.

More refined noninvasive tests such as Fibroscan, Hepascore, FibroMeter, FibroTest, or ELF could be used as second tier tests to further characterize patients, although this sequential approach needs prospective validation. The development and validation of such noninvasive algorithms will likely limit the need for liver biopsy in a selected few in the near future.

## Figures and Tables

**Table 1:** Noninvasive fibrosis serum tests and scores to diagnose nonalcoholic steatohepatitis (NASH) and/or stage fibrosis in patients with nonalcoholic fatty liver disease (NAFLD) [12].

Test	Variables	NAFLD stage assessed	Cut-off	AUROC
AST : ALT ratio	ALT and AST serum levels	F4	1	NA

CK-18	Cytokeratin 18 fragments	NAFLD NASH Fibrosis (any degree)	NA	0.77 0.65–0.83 0.68

Ferritin	Serum ferritin	NASH/≥F2 ≥F3	1.5 ULN 2.5 ULN	0.57 NA

NASH diagnostic	CK-18 fragments, adiponectin, and resistin	NASH	0.43	0.70–0.85

NASH diagnostic panel	Gender, BMI, diabetes, triglycerides, and CK-18 (total levels and fragments)	NASH	NA	0.81

NashTest	Age, gender, weight, height, bilirubin, GGT, *α*2-macroglobulin, apolipoprotein A1, haptoglobin, AST, serum glucose, triglycerides, and cholesterol	No NASH Borderline NASH NASH	NA	NA NA 0.69–0.83

NAFIC scoring system	Ferritin ≥200 or ≥300 ng/mL (F or M) = 1 Serum fasting insulin ≥10 *μ*U/mL = 1 Serum type IV collagen 7s ≥5.0 ng/mL = 2	NASH	2	0.78–0.85

Modified NAFIC scoring system	Ferritin ≥200 or ≥300 ng/mL (F or M) = 1 Serum fasting insulin ≥10 < 15 *μ*U/mL = 1 Serum fasting insulin ≥15 = 2 Serum type IV collagen 7s ≥5.0 ng/mL = 2	NASH	2	0.80

PIIINP	Terminal peptide of procollagen III levels	NASH ≥F3	6.6 ng/mL (low cut-off) 11 ng/mL (high cut-off)	0.82 0.84

APRI	AST, PLT	≥F2	0.45 (low cut-off) 1.5 (high cut-off)	0.62–0.94

BARD	BMI, AST/ALT ratio, and diabetes	≥F3	≥2	0.80

BARDI	BMI, AST/ALT ratio, diabetes, and INR	≥F3	≥3	0.88

NAFLD fibrosis score	Age, BMI, diabetes. AST, ALT, platelet count, and albumin	≥F3	−1.45 (low cut-off) 0.67 (high cut-off)	0.82–0.88

FIB-4	Age, AST, ALT, and platelet count	≥F3	1.3–1.92 (low cut-off) 3.25 (high cut-off)	0.87, 0.88

Hepascore	Age, sex, bilirubin, GGT, *α*2-macroglobulin, and hyaluronic acid	≥F2 ≥F3 F4	0.44 0.37 0.7	0.73 0.81 0.9

ELF test	Hyaluronic acid (HA), PIIINP, and tissue inhibitor of metalloproteinase 1 (TIMP-1).	≥F2 ≥F3 F4	8.5–10.18 10.35 (9.33–10.51) 9.35–11.3	0.82 0.90 0.87

FibroTest	Haptoglobin, *α*2-macroglobulin, apolipoprotein-A, bilirubin, and GGT	≥F3	0.3 (low cut-off) 0.7 (high cut-off)	0.88

FibroMeter	Age, weight, glucose, AST, ALT, PLT, and ferritin	≥F2 ≥F3 F4	F3: 0.61 (low cut-off) 0.71 (high cut-off)	0.94 0.93 0.9

ALT: alanine aminotransferase; AST: aspartate aminotransferase; AUROC: area under receiver operator characteristic curve; CK-18: cytokeratin 18; NA: not available; BMI: body mass index; GGT: *γ*-glutamyl transpeptidase; PLT: platelets; PIIINP: terminal peptide of procollagen III; APRI: aspartate aminotransferase to platelets ratio; INR: international normalized ratio; ELF: enhanced liver fibrosis; ULN: upper limit of normal.
